# Significant Interrelations among Serum Annexin A1, Soluble Receptor for Advanced Glycation End Products (sRAGE) and rs2070600 in Chronic Obstructive Pulmonary Disease

**DOI:** 10.3390/biology11121707

**Published:** 2022-11-25

**Authors:** Amira A. Kamel, Maiada K. Hashem, Ebtsam S. AbdulKareem, Amal H. Ali, Ekram Abdel-Rahman Mahmoud, Alaa S. Abd-Elkader, Hebatallah Abdellatif, Alzahra Abdelbadea, Nessren M. Abdel-Rady, Mona Gamal E. Al Anany, Marwa A. Dahpy

**Affiliations:** 1 Department of Medical Biochemistry, and Molecular Biology, Faculty of Medicine, Assiut University, Assiut 71515, Egypt; 2 Chest Department, Faculty of Medicine, Assiut University, Assiut 71526, Egypt; 3 Chest Department, Faculty of Medicine, Aswan University, Aswan 81528, Egypt; 4 Microbiology and Immunology Department, Faculty of Medicine, Aswan University, Aswan 81528, Egypt; 5 Microbiology and Immunology Department, Faculty of Medicine, Sohag University, Sohag 82524, Egypt; 6 Clinical Pathology Department, Faculty of Medicine, Assiut University, Assiut 71526, Egypt; 7 Clinical Pathology Department, Faculty of Medicine, Aswan University, Aswan 81528, Egypt; 8 Medical Biochemistry, and Molecular Biology, Faculty of Medicine for Girls, Al-Azhar University, Cairo 11651, Egypt; 9 Medical Physiology Department, Faculty of Medicine, Assiut University, Assiut 71526, Egypt; 10 Medical Physiology Department, Sphinx University, New-Assiut 71515, Egypt; 11 Physiology Department, Faculty of Medicine for Girls, Al-Azhar University, Cairo 11651, Egypt; 12 Department of Medical Biochemistry and Molecular Biology, Armed Forces College of Medicine (AFCM), Cairo 11774, Egypt

**Keywords:** annexin A1, sRAGE, rs2070600, COPD

## Abstract

**Simple Summary:**

Considering that COPD is a major cause of death and morbidity, this study therefore aimed to find circulating biomarkers for COPD that could help with early diagnosis, predict exacerbation and understand the pathogenesis of the disease. The rs2070600 gene polymorphism, serum sRAGE, annexin A1, GSH and MDA levels were determined. The rs2070600 gene polymorphism has a strong association with COPD, as revealed by genotyping and allelic frequency distribution. sRAGE serum levels were significantly lower, while annexin A1 levels were much greater in COPD patients as compared to controls. The GA genotype was most distributed in COPD, and it was strongly linked to lower serum sRAGE levels. Serum sRAGE and annexin A1 may be considered potential diagnostic tools for COPD. Through impacts on GSH and MDA levels that alter the release of inflammatory factors and, therefore, lung damage, it is possible to explain the relationship between annexin A1, sRAGE, and COPD.

**Abstract:**

Chronic obstructive pulmonary disease (COPD) is a major cause of death and morbidity; it may be accompanied by oxidative stress and inflammation with or without underlying genetic etiology. Finding circulating biomarkers for COPD that can help early diagnosis and predict exacerbation and association with respiratory functions has been challenging. There were 40 healthy participants and 60 COPD patients in this research. The rs2070600 gene variant was examined by PCR-RFLP. Circulating sRAGE and annexin A1 levels were determined by ELISA. GSH and MDA were determined by spectrophotometry. In COPD patients, sRAGE serum levels were substantially lower, but conversely, annexin A1 levels were much greater than in controls. The rs2070600 gene polymorphism’s strong association with COPD was demonstrated by genotyping and allelic frequency distribution. The GA genotype was most distributed in COPD, and it was strongly linked to lower serum sRAGE levels. The interrelation between annexin A1, sRAGE, and COPD could be explained through effects on inflammatory mediators’ pathways. The rs2070600 gene polymorphism was found to significantly enhance the risk of COPD. Serum sRAGE and annexin A1 may be considered potential diagnostic tools for COPD. Through impacts on GSH and MDA levels that alter the release of inflammatory factors and, therefore, lung damage, it is possible to explain the relationship between annexin A1, sRAGE, and COPD.

## 1. Introduction

With chronic bronchitis, emphysema, and narrowing of the airways as its hallmarks, chronic obstructive pulmonary disease (COPD) is a progressive heterogeneous respiratory disease that makes breathing difficult [1]. Globally, chronic obstructive pulmonary disease (COPD) is one of the leading causes of morbidity, mortality, and the use of medical services [2]. It is characterized by sputum production and airflow obstruction, as well as respiratory symptoms (such as dyspnea, coughing, and dyspnea) [3,4]. The underlying cause of COPD is typically chronic inflammation brought on by a number of environmental and/or genetic causes [5,6].

In COPD, protein biomarker panels evaluated in blood samples have been researched; these panels may be connected with significant clinical outcomes and may be utilized to identify individuals with asthma severity or exacerbations [7].

A member of the immunoglobulin superfamily of receptors, RAGE functions as both a pattern recognition receptor and an alarming receptor [8]. Human RAGE is a multi-ligand transmembrane protein receptor [9] that is modestly expressed in a variety of organs and substantially expressed in the lungs throughout life. The gene encoding human RAGE is found on chromosome 6p21. It is made up of three primary regions: the cytoplasmic, the transmembrane, and the extracellular area [10,11].

Blood and other bodily fluids have two primary soluble versions of the RAGE receptor that are both devoid of the transmembrane and cytoplasmic domains [12]. Known together as soluble RAGE (sRAGE), these two RAGE isoforms [13] are increasingly linked to host defense against infections, inflammation, cardiometabolic illnesses, and age-related disease [14]. As a decoy receptor, sRAGE removes circulating AGEs and keeps them from attaching to membrane-bound RAGE, protecting lung tissue from damage [7].

Systemic soluble receptors for advanced glycation end-products (sRAGE) levels have been linked to emphysema, disease progression, and a loss in lung function in COPD patients, according to many studies [15,16].

The altered circulating levels of sRAGE, which binds to RAGE ligands to function as a decoy to interfere with the RAGE signaling pathway, are linked to the AGER polymorphisms [17]. By changing the 82nd amino acid of RAGE from glycine to serine, the rs2070600 SNP boosts the glycation rate of one of the two glycation sites at the ligand-binding domain, which in turn increases the ligand-binding capability of RAGE [15].

An additional marker is annexin A1, a calcium-dependent phospholipid-binding protein that is crucial for the cell’s anti-inflammatory response [18,19]. It restricts the early stages of inflammation [20], including the synthesis of inflammatory mediators and the replacement of white blood cells [15].

In this case–control study, we focused on assessing the role and exploring the relationships between circulating sRAGE, annexin A1 levels and rs2070600 with respiratory function and other clinical aspects of COPD, aiming to identify early and easily accessible diagnostic tools for COPD and to help reduce its progression and exacerbation.

## 2. Subjects and Methods

### 2.1. Subjects and Anthropometric Parameters

Case–control research is what this study entails. Along with 40 perfectly healthy controls who were age- and sex-matched, 60 patients with COPD were recruited from the Assiut University Hospital’s Chest Department and outpatient clinics. This was done in collaboration with the Department of Medical Biochemistry and Molecular Biology in the faculty of medicine. For their part in the study, each subject gave their written informed consent. The Institutional Review Board/Medical Ethics of the Faculty of Medicine at Assiut University examined and approved the study protocol (IRB 17300767), and all study procedures were carried out in accordance with the Helsinki declaration.

Routine physical and clinical exams of all subjects were performed, including measures of body weight (kg), waist circumference (cm), height (cm), body mass index (BMI), and blood pressure (mm Hg).

According to the Global Initiative for Chronic Obstructive Lung Disease (GOLD) staging guidelines, patients were given a COPD diagnosis [21]. A measurement of FEV1/FVC < 70% of the predicted value and/or FEV1 < 80% of the predicted value, and having gone at least 4 weeks without showing any signs of a respiratory infection or an acute exacerbation.

During the examination, a smoking habit assessment interview was conducted. Subjects who reported regularly and currently smoking cigarettes were considered to be current smokers. Subjects who had given up smoking for at least a year were considered ex-smokers. Subjects who said they had never smoked were referred to as non-smokers. The study excluded those having a history of cancer, cardiovascular disease, dementia, or other systemic illnesses, as well as asthma or other airway diseases.

### 2.2. Blood Sample Collection and General Biochemical Marker Assays

All participants who were included had their venous blood drawn in the amount of 5 mL. For DNA extraction, CBC, and HbA1c assays, 1 mL of blood was drawn into tubes containing EDTA. Through centrifugation at 3000 rpm for 10 min, the remaining 4 mL of blood was utilized to separate the serum. The samples were maintained at −20 °C.

Randox’s enzymatic glucose kit was used to measure serum glucose levels (Catalog No. GL364, Randox Laboratories Limited, Crumlin, County Antrim, UK). Spectrum Diagnostics Creatinine Reagent was used to test serum creatinine (Catalog No. 235002). Bio-Diagnostics reagent was used to test serum urea (Catalog No. 2110). A fresh EDTA blood sample was used for the Spectrum Diagnostics turbidimetric immunoassay to estimate the levels of glycated haemoglobin (HbA1c) (Catalog No. 602 001-I). Liver function was assessed using Bio Diagnostic-provided colorimetric kits (Catalog No. AL 10 31, AS 10 61).

### 2.3. Determination of Serum sRAGE, Annexin A1, GSH and MDA Concentrations

Serum sRAGE and annexin A1 levels were measured by ELISA kits supplied by SinoGene clon (Catalog No. SG-10679) and Glory Science Co., Ltd., Hongkong, China (ANX-A1), respectively.

Malondialdehyde (MDA) and reduced glutathione (GSH) serum levels were tested using kits provided by Bio Diagnostic—Egypt (CAT. Nos. MD 25 29 and GR 25 11), respectively.

### 2.4. DNA Extraction

The QIAamp DNA mini extraction kit, Catalog No. 51104, offered by Qiagen Company, was used to extract DNA from the peripheral whole blood samples in accordance with the manufacturer’s instructions. A Nanodrop was used to verify the concentration and purity of the DNA that was extracted (Epoch, Biotek, Winooski, VT, USA). DNA absorbance at 260 nm was used to determine the concentration of DNA samples. The 260/280 ratio of DNA samples was regarded as pure if it was 1.8–2. Until genotyping, the DNA was stored at −20 °C.

### 2.5. Genotyping

The RAGE intron of the rs2070600 mutation was genotyped using PCR-RFLP amplification following MfeI enzymatic digestion (Fermentas, Vilnius, Lithuania). The following primers were utilized: forward primer, 5′-GTA AGC GGG GCT CCT GTT GCA-3; reverse primer, 5′-GGC CAA GGC TGG GGT TGA AGG-3″ (5).

By electrophoresis on 3% agarose gel, all of the digestion products were examined. For quality assurance, we sequenced 5% of the DNA samples directly for each SNP; no discrepancies were found. Without knowing the grouping design, the findings were read and analyzed in a blinded way.

### 2.6. Statistical Analysis

The IBM SPSS software version 20.0 (SPSS Inc., Chicago, IL, USA) and Graph Pad Prism 7 software (San Diego, CA, USA) were used to conduct all statistical analyses. The means ± standard deviations were used to convey quantitative data (SD). Frequency and percentage were used to convey qualitative data. Independent-samples *t*-tests, Student’s *t*-tests, or Mann–Whitney tests were employed for comparing two means, if applicable, with a significance level of *p* < 0.05. Using a χ^2^ test, the Hardy–Weinberg equilibrium was evaluated. Fisher’s exact tests or chi-squared tests were used to analyze the genotype and allele distributions within groups. As a measure of how strongly the examined genotypes were associated with COPD, odds ratios (OR) and 95% confidence intervals (CI) were determined independently. When comparing more than two means, a one-way analysis of variance (ANOVA) and post-hoc tests were utilized. Spearman’s correlation coefficient was used for the correlation analysis. The cut-off value was established using MedCalc software version 14 using the receiver operating characteristic (ROC) curve of blood levels of sRAGE and annexin A1 concentrations to assess their diagnostic performance between groups.

## 3. Results

### 3.1. Baseline Characteristics

Table 1 displays the participants’ demographic and clinical characteristics. The average age of the patients (50 men and 10 women) was 62.65 ± 6.49 years, as opposed to 61.45 ± 5.72 years for the control group (32 men and 8 women). Regarding age (*p* = 0.345), gender (*p* = 0.671), and BMI (*p* = 0.067), there were no discernible differences between the COPD patient and control groups. There were more ex-smokers in the COPD group in terms of smoking status (65% vs. 17.5%, *p* < 0.001).

In terms of exacerbations, 68.3% of COPD patients had had two or more in the past, compared to 31.7% who had experienced none or one in the past. A total of 55% of COPD patients had no additional concomitant conditions, followed by those with hypertension (23.3%), diabetes (15%), and cardiovascular disease (6.7%). In comparison to the control group, the COPD group’s forced expiratory volume in 1 s (FEV1) and FEV1/FVC were considerably lower (all *p* < 0.001). A total of 6.7% of patients were in stage I, 43.3% were in stage II, 41.7% were in stage III, and 8.3% were in stage IV when using the GOLD staging system.

The laboratory parameters of the participants are displayed in Table 2. In comparison to controls, patients had considerably higher levels of PaCO_2_ and HCO_3_ and significantly lower levels of PaO_2_ and SaO_2_. Other laboratory parameters compared between the two research groups revealed no differences.

### 3.2. Analysis of RAGE rs2070600 Gene Polymorphism

Following enzymatic digestion, the following fragments were discovered: 276 bp for the wild-type alleles and 64 and 212 bp for the minor allele. The analysis of the RAGE rs2070600 variants is shown in Table 3. For both COPD patients and controls, the distribution of the genotypes of the investigated SNP followed the Hardy–Weinberg equilibrium.

Regarding the RAGE gene’s genotype variants, COPD patients had a distribution of GG, GA, and AA genotype variants of 28.3%, 55%, and 16.7%, respectively, whereas controls had a distribution of 62.5%, 25%, and 12.5%. As a result, controls had a higher prevalence of the GG genotype (62.5%) than COPD patients (55%), who had a higher prevalence of the GA genotype. The RAGE polymorphism’s G vs. A allele frequencies were 75 versus 25% among control persons and 55.8% against 44.2% among patients (*p* = 0.006). In comparison to controls, patients had substantially increased RAGE GA genotype and G allele frequencies (OR = 3.667, 95% CI: 1.524–8.822, *p* = 0.003 and OR = 0.421, 95% CI: 0.226–0.784, *p* = 0.006, respectively).

To find out whether the prevalence of certain genotypes or alleles was linked to tobacco exposure, the genotype and allele distributions between study participants who were smokers (both current and ex-smokers) and non-smokers were investigated and compared. Table 4 presents the findings. In both current and ex-smokers, COPD patients had a considerably greater frequency of the RAGE GA genotype than did controls (OR = 3.707, 95% CI: 1.046–13.134, *p* = 0.035). In contrast, there was no statistically significant difference in the frequency of the RAGE GA genotype among controls and COPD patients who did not smoke (OR = 1.583, 95% CI: 0.230–10.904, *p* = 0.639). Additionally, there was no statistically significant difference between the COPD group and controls for either smokers (*p* = 0.60) or non-smokers (*p* = 0.172) in terms of the G allele or A allele of the RAGE G82S polymorphism.

### 3.3. Analysis of Serum sRAGE, Annexin A1 Levels and Inflammatory Mediators

**Figure 1:**
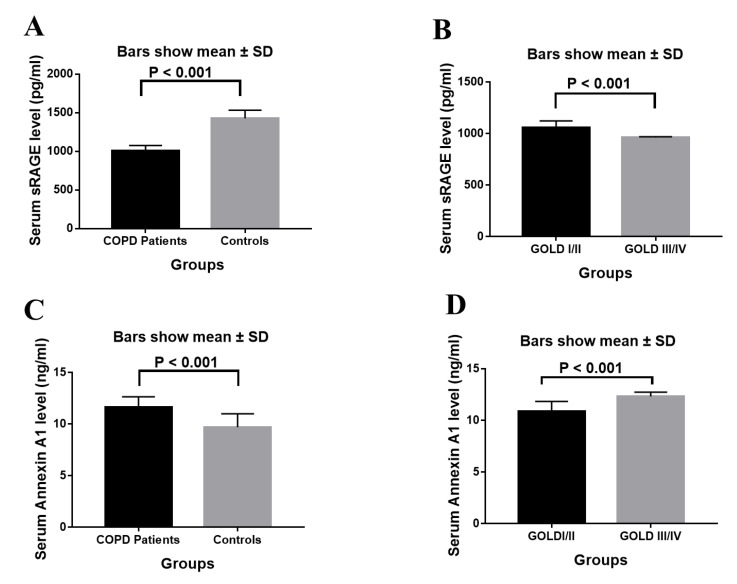
(**A**). Serum level of sRAGE in COPD patients and controls. (**B**). Serum level of sRAGE in COPD patients stratified according to disease severity (GOLD stages). (**C**). Serum level of annexin A1 in COPD patients and controls. (**D**). Serum level of annexin A1 in COPD patients and controls. Data are presented as mean ± SD. Abbreviations: COPD; chronic obstructive pulmonary disease, GOLD, Global Initiative for Chronic Obstructive Lung Disease, sRAGE, soluble receptor for advanced glycation end products.

Since the average sRAGE levels in the COPD patients were 1008.78 ± 67.50 pg/mL and 1428.32 ± 103.49 pg/mL in the controls, the serum sRAGE levels in the COPD patients were considerably lower than those in the controls (*p* < 0.001). (Table 5 and Figure 1). Additionally, the serum sRAGE levels in COPD patients were considerably lower in GOLD stages III and IV (960.57 ± 8.74 vs. 1057 ± 66.21, respectively, *p* < 0.001) than in stages I and II.

Serum annexin A1 levels in COPD patients were considerably higher (11.608 ± 1.03) than in controls (9.697 ± 1.3, *p* < 0.001) (Table 5 and Figure 1). In addition, COPD patients in GOLD stages III and IV (11.81 ± 1.15) had substantially higher serum levels of annexin A1 than COPD patients in GOLD stages I and II (11.41 ± 0.87, *p* = 0.002).

In terms of inflammatory mediators, serum levels of GSH were significantly higher in healthy controls (41.91 ± 3.79) than in COPD cases (16.33 ± 2.76, *p* < 0.001). In contrast, MDA levels were significantly higher in COPD (6.88 ± 1.72) than in healthy controls (3.39 ± 0.71, *p* < 0.001).

### 3.4. Association between sRAGE, Annexin A1 Levels and RAGE rs2070600 Polymorphism

Regarding RAGE rs2070600 polymorphism (Table 6), patients with COPD who had the GA genotype had lower levels of sRAGE (994.3 ± 39.54 pg/mL) compared with those carrying the GG genotype (1059.88± 95.59 pg/mL; *p* = 0.001). Additionally, the COPD patients bearing the AA genotype had lower sRAGE levels (969.7 ± 25.83 pg/mL) compared with those carrying the GG genotype (1059.88 ± 95.59 pg/mL; *p* < 0.001). Serum levels of sRAGE were lower in the AA genotype (969.7 ± 25.83 pg/mL) compared to the GA genotype (994.3 ± 39.54; *p* = 0.257) but did not reach a significance level. Concerning serum levels of annexin A1 in COPD patients, there was no discernible difference between the three genotypes.

The participants in the control group who had the rs2070600 GA genotype had considerably lower blood sRAGE levels (1407.1 ± 32.49 pg/mL) than those who had the GG genotype (1473.68 ± 40.86 pg/mL; *p* = 0.003). Serum sRAGE levels were also significantly lower in the AA genotype (1202.8 ± 131.83 pg/mL) than in the subjects with the GA genotype (1407.1 ± 32.49 pg/mL; *p* < 0.001) and in the AA genotype (1202.8 ± 131.83 pg/mL) compared to the GG genotype (1473.68 ± 40.86 pg/mL; *p* < 0.001). As regards annexin A1, results did not reveal a significant difference among the three genotypes in control subjects.

### 3.5. Diagnostic Performance of sRAGE and Annexin A1

**Figure 2:**
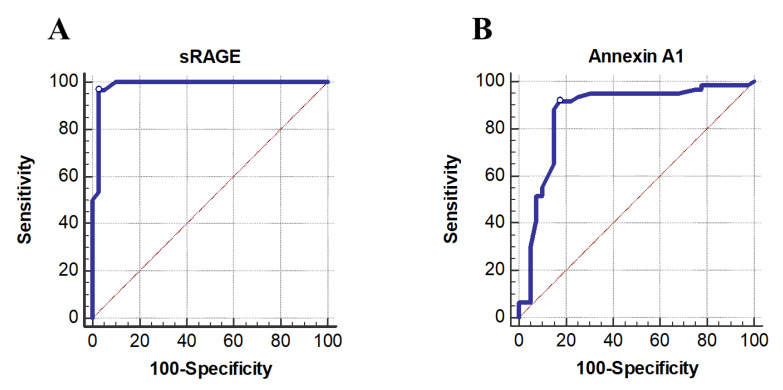
Predictive performances (ROC curves) of (**A**). sRAGE and (**B**). Annexin A1serum level for discrimination between COPD and control groups. Abbreviations: ROC curve, receiver operating characteristic curve; sRAGE, soluble receptor for advanced glycation end products.

The ability of blood levels of sRAGE and annexin A1 to distinguish between COPD patients and controls was assessed using an ROC curve (Figure 2).

Interestingly, sRAGE demonstrated the largest areas under the curve (AUC) and the strongest predictive value for the development of COPD, followed by annexin A1. According to the maximum of the Youden Index, the sRAGE had an AUC of 0.986, and its cut-off value that best predicted COPD was 1079 pg/mL (sensitivity = 96.67%; specificity = 97.5%, PPV = 98.3, NPV = 95.1, *p* < 0.001). As regards annexin A1, the results suggested a cut-off value of 10.7 ng/mL and AUC of 0.868 (91.67% sensitive and 82.50% specific, PPV = 88.7%, NPV = 86.8%, *p* < 0.001) between the COPD patient group and the control group.

### 3.6. Correlations between sRAGE, Annexin A1 and FEV1 Predicted

**Figure 3:**
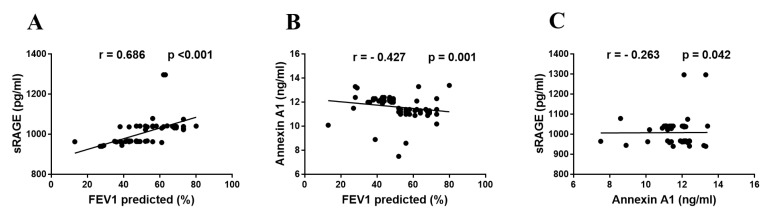
Correlations in COPD patients between (**A**). sRAGE and FEV1; (**B**). annexin A1 and FEV1; (**C**). sRAGE and annexin A1. Abbreviations: FEV1, forced expiratory volume in the first second; sRAGE, soluble receptor for advanced glycation end products.

In COPD patients, our results show a significant positive correlation between serum sRAGE level and FEV1 predicted (r = 686, *p* < 0.001), a significant negative correlation between serum annexin A1 level and FEV1 predicted (r = −0.427, *p* = 0.001) and a significant negative correlation between serum sRAGE level and serum annexin A1 level (r = −0.263, *p* = 0.042), as shown in Figure 3.

## 4. Discussion

Systemic inflammation, which can be brought on by a variety of factors, including airway inflammation and smoking, is frequently present in chronic obstructive pulmonary disease (COPD) patients.

As the forced expiratory volume in one second (FEV1) and the FEV1/FVC were considerably lower in the COPD group than in the control group (all *p* < 0.001), the findings of the current study suggested that patients with COPD had impaired lung function.

Additionally, the current study’s findings support the link between sRAGE and COPD. In addition to a significant positive correlation between serum sRAGE levels and FEV1 predicted in COPD patients, the serum sRAGE levels were significantly correlated with the presence and stage of COPD. Its levels in COPD patients were significantly lower than those in controls, and they were significantly lower in GOLD stage III and IV than in GOLD stage I and II.

Since the soluble receptor for advanced glycation end-products (sRAGE) is thought to act as a sink for pro-inflammatory RAGE ligands, it has traditionally been linked to a reduction in the risk of inflammation-related stress and illness [22].

Our findings are in accordance with [1,23], who found that patients with clinically stable COPD had lower plasma sRAGE levels, and sRAGE is a strong predictor of FEV1 and has a favorable connection with it.

Numerous cross-sectional investigations revealed a reliable relationship between sRAGE and other features of COPD. For instance, Cockayne and colleagues [24] and Iwamoto and colleagues [25] demonstrated that systemic RAGE levels correlate with smoking and/or COPD disease status. They also discovered that plasma levels of sRAGE in patients with COPD and those who had overlapping COPD and asthma were lower in these patients than in patients with asthma and in healthy control subjects. The drop in FEV1/FVC was also linked to and predicted by reduced sRAGE levels.

Several crucial metabolic processes during inflammation are modulated by annexin A1 [26]. However, nothing is understood regarding its function in COPD airway fibrosis [27].

Regarding annexin A1, our present study showed that COPD patients had considerably higher blood levels of annexin A1 than did controls and that these levels were also significantly higher in COPD patients in GOLD stages III and IV than in GOLD stages I and II. A significant negative correlation was found between serum annexin A1 level and FEV1 predicted, and a significant negative correlation was also found between serum sRAGE levels and serum annexin A1 levels. These findings are in accordance with [27], who concluded that lung fibroblast activity is impacted by elevated annexin A1 expression in COPD patients.

To test the diagnostic utility of the studied biomarker, an ROC curve was created. sRAGE showed the highest predictive value for COPD development, followed by annexin A1. In particular, for sRAGE (sensitivity = 96.6%; specificity = 0.975%, *p* < 0.001), regarding annexin A1, the results suggested a cut-off value of 10.7 ng/mL with 91.67% sensitivity and 82.50% specificity between the COPD patient group and the control group.

The interrelation between AnxA1, sRAGE and COPD could be explained through effects on inflammatory mediator pathways, as our results found significantly higher levels of serum GSH in healthy controls than in COPD cases, which is related to elevated sRAGE levels. Conversely, MDA levels were noticeably greater in COPD patients compared to healthy controls.

Regarding the RAGE gene single nucleotide variant rs2070600, our results provide evidence that this SNP is linked to a higher risk of COPD development. Comparing COPD patients to controls, it was shown that the GA genotype and G allele were substantially more common. In contrast to controls, where the GG genotype predominates (62.5%), COPD patients have a GA genotype that is more prevalent (55%) in them. The G versus A allele frequencies of the RAGE G82A polymorphism were 55.8% versus 44.2% among the patients and 75% versus 25% among the control subjects (*p* = 0.006). Genotype and allele distributions were not significantly different among smokers and non-smokers

The rs2070600 GA genotype-carrying COPD patients had lower blood levels of sRAGE. Additionally, the sRAGE levels were lower in COPD patients with the AA genotype. Concerning serum levels of annexin A1, there was no significant difference among the three genotypes in each of the COPD patients and control subjects.

The participants in the control group who had the rs2070600 GA genotype had considerably lower blood levels of sRAGE than those who had the GG genotype. Serum sRAGE levels were also significantly lower in the AA genotype than in subjects with the GA genotype and in the AA genotype compared to the GG genotype.

Our findings are consistent with those of Li et al., who also offer proof that a genetic variant of the RAGE gene (G82S) is linked to the potential development of COPD [10].

Our findings confirmed the long-established effects of sRAGE, which have been investigated as a marker of disease risk and unfavorable outcomes. Its potential association with the etiology and grades of COPD may be due to its effect on inflammatory markers (GSH, MDA), and soluble RAGE has been shown to be protective against the pathogenesis of COPD [1] by blocking the traditional RAGE signaling pathway.

A glycine-to-serine amino acid substitution at position 82 of RAGE results in the single nucleotide polymorphism rs2070600, which raises the rate of glycation at one of the two glycation sites at the ligand-binding domain and therefore raises the ligand-binding capability of RAGE [15].

Smoking cigarettes increases the chance of developing a number of illnesses, including COPD and cardiovascular disease [28,29]. Interrelation between AnxA1, sRAGE and COPD could be explained through effects on inflammatory pathways, GSH, and MDA which are interrelated to the NFkB/Akt signaling pathway. The results of Yunzi et al. [30] support our results as they found that AnxA1peptide increases Akt phosphorylation levels, suppresses p-NF-B (p65) expression, and lessens the production of inflammatory mediators and lung damage.

This study has two limitations. First, the numbers of COPD cases and healthy controls were insufficient. Second, only one SNP in the RAGE gene was used for this study. Therefore, further investigations between other SNPs of the RAGE gene and COPD with a larger sample size are required.

## 5. Conclusions

The rs2070600 gene polymorphism’s genotyping and allelic frequency distribution showed a strong correlation with an increased risk of COPD. The GA genotype was most distributed in COPD, and it was closely related to decreased serum levels of sRAGE.

Serum sRAGE levels are considered a marker of COPD disease. Its levels are considerably lower in COPD patients compared to control persons. On the contrary, circulating annexin A1 levels were significantly greater in COPD than in controls.

The interrelation between AnxA1, sRAGE and COPD could be explained through their effects on inflammatory mediator pathways, including through GSH and MDA levels, which affect the release of inflammatory factors and, subsequently, lung injury.

This finding provides insights into the etiology and pathogenesis of COPD, which may help create novel therapies and improve the care of COPD patients.

## Figures and Tables

**Table 1:** Demographic and clinical characteristics of COPD patients and controls.

	COPD Patients (*n* = 60)	Controls (*n* = 40)	*p*-Value
Age (Year)			
Mean ± SD	62.65 ± 6.49	61.45 ± 5.72	0.345
Sex			
Male *n* (%)	50 (83.3%)	32 (80%)	0.671
Female *n* (%)	10 (16.7%)	8 (20%)	
BMI (kg/m^2^)			
Mean ± SD	26.42 ± 6.72	28.362 ± 6.29	0.067
Smoking Status *n* (%)			
Current	15 (25)	8 (20)	**<0.001**
Ex	39 (65)	7 (17.5)	
None	6 (10)	25 (62.5)	
Comorbidity *n* (%)		NA	
DM	9 (15)		
HTN	14 (23.3)		
Cardiovascular	4 (6.7)		
None	33 (55)		
Exacerbations *n* (%)		NA	
None or One	19 (31.7)		
Two or More	41 (68.3)		
History *n* (%)		NA	
Cough	8 (13.3)		
Dyspnea	9 (15)		
Cough, Dyspnea	31 (51.7)		
Cough, Dyspnea, Wheezes	12 (20)		
FEV1/FVC (%) post-bronchodilator			
Mean ± SD	53.28 ± 8.1721	77.05 ± 4.37	**<0.001**
FEV1 (%) Predicted			
Mean ± SD	51.48 ± 13.76	85.0 ± 2.85	**<0.001**
GOLD staging *n* (%)		NA	
Mild	4 (6.7)		
Moderate	26 (43.3)		
Severe	25 (41.7)		
Very severe	5 (8.3)		

Data are presented as the mean ± SD or *n* (%) unless otherwise stated. The bold value indicates statistical significance. Notes: Severity of COPD was graded according to GOLD. GOLD I, mild, FEV1% predicted ≥ 80%; GOLD II, moderate, FEV1% predicted 50–79%; GOLD III, severe, FEV1% predicted 30–49; GOLD IV, very severe, FEV1% predicted <30%. Abbreviations: BMI, body mass index; COPD, chronic obstructive pulmonary disease, DM, diabetes mellitus; FEV1, forced expiratory volume in the first second; FVC, forced vital capacity, GOLD, Global Initiative for Chronic Obstructive Lung Disease; HTN, hypertension.

**Table 2:** Laboratory parameters of COPD patients and controls.

	COPD Patients (*n* = 60)	Controls (*n* = 40)	*p*-Value
WBC (10 × 3/µL)			
Mean ± SD	7.41 ± 1.9	7.78 ± 1.65	0.26
Hb (g/dL)			
Mean ± SD	13.11 ± 1.49	12.6 ± 1.54	0.167
HCT (%)			
Mean ± SD	42.75 ± 4.36	41.21 ± 4.52	0.135
PLT (10 × 3/µL)			
Mean ± SD	209.05 ± 55.49	206.27 ± 54.71	0.74
Urea (mmol/L)			
Mean ± SD	6.8 ± 2.32	6.55 ± 1.98	0.708
Creatinine (µmol/L)			
Mean ± SD	79.7± 24.88	77.57 ± 23.59	0.554
Bilirubin (mg/dL)			
Mean ± SD	0.66 ± 0.26	0.65 ± 0.23	0.792
Total serum Protein (g/dL)			
Mean ± SD	6.3 ± 0.57	6.52 ± 0.64	0.149
Albumin (g/dL)			
Mean ± SD	3.41 ± 0.24	3.46 ± 0.21	0.331
ALT (U/L)			
Mean ± SD	23.1 ± 8.04	22.3 ± 7.28	0.565
AST (U/L)			
Mean ± SD	32.11 ± 7.1	31.32 ± 6.47	0.367
ESR (mm/h)			
Mean ± SD	17.7 ± 12.54	15.95 ± 10.61	0.37
pH			
Mean ± SD	7.415 ± 0.023	7.409 ± 0.022	0.17
PaCO_2_ (mmHg)			
Mean ± SD	55.383 ± 6.86	41.0 ± 3.70	**<0.001**
PaO_2_ (mmHg)			
Mean ± SD	75.9 ± 7.04	80.07 ± 4.72	**0.003**
SaO_2_ (%)			
Mean ± SD	94.8 ± 1.18	95.62 ± 1.19	**0.001**
HCO_3_ (mEq/L)			
Mean ± SD	37.53 ± 4.81	25.85 ± 2.39	**<0.001**

Data are presented as the mean ± SD or *n* (%) unless otherwise stated. The bold value indicates statistical significance. Abbreviations: ALT, alanine transaminase; AST, aspartate transaminase; ESR, erythrocyte sedimentation rate; Hb, hemoglobin; HCO_3_, bicarbonate; HCT, hematocrit; PaCO_2_, partial pressure of carbon dioxide; PaO_2_, partial pressure of oxygen; PLT, platelet; SaO_2_, arterial oxygen saturation; WBC, white blood cell.

**Table 3:** Genotype and allele frequencies of RAGE rs2070600 gene polymorphism in COPD patients and controls.

	COPD Patients (*n* = 60)	Controls (*n* = 40)	OR (95% CI)	*p*-Value
**Genotype**				
**GG**	17 (28.3%)	25 (62.5%)	0.237 (0.101–0.556)	0.001
**GA**	33 (55%)	10 (25%)	3.667 (1.524–8.822)	0.003
**AA**	10 (16.7%)	5 (12.5%)	1.4 (0.44–4.453)	0.568
**Allele**				
**G**	67 (55.8%)	60 (75%)	0.421 (0.226–0.784)	0.006
**A**	53 (44.2%)	20 (25%)		

Data are presented as number (%). The bold value indicates statistical significance. Abbreviations: COPD, chronic obstructive pulmonary disease; OR, odds ratio; RAGE, receptor for advanced glycation end products.

**Table 4:** Genotype and allele frequencies of RAGE rs2070600 gene polymorphism in COPD patients and controls and corresponding ORs for COPD by smoking status.

	Smokers (Current and Ex) (*n* = 69)				Non-Smokers (*n* = 31)			
Genotype	COPD Patients (*n* = 54)	Controls (*n* = 15)	OR (95% CI)	*p*-Value	COPD Patients (*n* = 6)	Controls (*n* = 25)	OR (95% CI)	*p*-Value
GG	14 (25.9%)	7 (46.7%)	0.40 (0.123–1.306)	0.122	3 (50%)	18 (72%)	0.389 (0.063–2.407)	0.301
GA	31 (57.4%)	4 (26.7%)	3.707 (1.046–13.134)	**0.035**	2 (33.3%)	6 (24%)	1.583 (0.230–10.904)	0.639
AA	9 (16.7%)	4 (26.7%)	0.550 (0.143–2.121)	0.381	1 (16.7%)	1 (4%)	4.800 (0.255–90.298)	0.257
Allele								
G	59 (54.6%)	18 (60%)	0.803 (0.353–1.828)	0.600	8 (66.7%)	42 (84%)	0.381 (0.092–1.574)	0.172
A	49 (45.4%)	12 (40%)			4 (33.3%)	8 (16%)		

Data are presented as number (%). The bold value indicates statistical significance. Abbreviations: COPD, chronic obstructive pulmonary disease; OR, odds ratio; RAGE, receptor for advanced glycation end products.

**Table 5:** Serum levels of sRAGE, annexin A1, MDA and GSH in COPD patients and controls.

	COPD Patients (*n* = 60)	Controls (*n* = 40)	*p*-Value	COPD Patients GOLD I, II (*n* = 30)	COPD Patients GOLD III, IV (*n* = 30)	*p*-Value
sRAGE pg/mL Mean ± SD	1008.78 ± 67.50	1428.32 ± 103.49	<0.001	1057.0 ± 66.21	960.57± 8.74	<0.001
Annexin A1 (ng/mL) Mean ± SD	11.608 ± 1.03	9.697 ± 1.3	<0.001	11.41 ± 0.87	11.81 ± 1.15	0.002
MDA (nmol/mL) Mean ± SD	6.88 ± 1.72	3.39 ± 0.71	<0.001	5.36 ± 0.78	8.4± 0.8	<0.001
GSH (mmol/L) Mean ± SD	16.33 ± 2.76	41.91 ± 3.79	<0.001	18.68 ± 1.02	13.98 ± 1.75	<0.001

Data are presented as the mean ± SD. The bold value indicates statistical significance. Abbreviations: COPD, chronic obstructive pulmonary disease; sRAGE, soluble receptor for advanced glycation end products.

**Table 6:** Serum levels of sRAGE in COPD patients and controls according to RAGE rs2070600 polymorphism.

COPD Patients							
	GG (*n* = 17)	GA (*n* = 33)	AA (*n* = 10)	P1	P2	P3	P4
sRAGE pg/mL							
Mean ± SD	1059.88 ± 95.59	994.3 ± 39.539	969.7 ± 25.83	<0.001	0.001	0.257	<0.001
Annexin A1 (ng/mL)							
Mean ± SD	11.56 ± 1.04	11.79 ± 0.75	11.1 ± 1.61	0.177	0.453	0.066	0.262
Controls							
	GG	GA	AA	P1	P2	P3	P4
	(*n* = 25)	(*n* = 10)	(*n* = 5)				
sRAGE pg/mL							
Mean ± SD	1473.68 ± 40.86	1407.1 ± 32.49	1202.8 ± 131.83	<0.001	0.003	<0.001	<0.001
Annexin A1 (ng/mL)							
Mean ± SD	9.8 ± 1.4	9.46 ± 1.33	9.64 ± 0.63	0.782	0.491	0.805	0.801

Data are presented as the mean ± SD. The bold value indicates statistical significance. P1 between groups, P2 between GG and GA, P3 between GA and AA, P4 between GG and AA. Abbreviations: COPD, chronic obstructive pulmonary disease; sRAGE, soluble receptor for advanced glycation end products.

## Data Availability

All related data and materials are available on request.
